# Oenological Characteristics of Fermented Apple Musts and Volatile Profile of Brandies Obtained from Different Apple Cultivars

**DOI:** 10.3390/biom10060853

**Published:** 2020-06-03

**Authors:** Magdalena Januszek, Paweł Satora, Tomasz Tarko

**Affiliations:** Department of Fermentation Technology and Microbiology, Faculty of Food Technology, University of Agriculture in Krakow, Balicka Street 122, 30-149 Krakow, Poland; pawel.satora@urk.edu.pl (P.S.); tomasz.tarko@urk.edu.pl (T.T.)

**Keywords:** apple brandies, volatile compounds, terpenes, esters, sensory analysis

## Abstract

Volatile profile of spirits is the most important factor, because it can contribute to pleasant flavor. The aim of the study was to determine the impact of dessert apple cultivar used for fermentation on the concentration of volatile compounds in apple spirits. SPME-GC-MS (solid-phase microextraction- gas chromatography- mass spectrometry) method enables the detection of 69 substances and GC-FID (gas chromatography - flame ionization detector) 31 compounds. Characteristic volatiles for brandies obtained from Topaz were limonene, myrcene, methyl valerate and 1,1-diethoxy-propane, from Rubin—β-citronellol and isopropyl acetate, Elise—limonene, myrcene benzyl acetate and isopropyl acetate, Szampion—β-citronellol, Idared—1,1-diethoxy-propane and Jonagored—ethyl *trans*-4-decanoate. Of the ten analyzed apple spirits, those obtained from Topaz, Rubin and Elise cultivars demonstrated the most diverse profile of volatile compounds. Moreover, their oenological parameters that are the most important in the production of alcoholic beverages were the most favorable. On the other hand, the content of sugars was relatively low in Elise must, while it was highest in Topaz must, which later on translated into differences in alcohol content. Brandies obtained from Gloster contained the smallest concentrations of esters and terpenes. Results of the sensory analysis showed that highest rated brandies were obtained from Topaz, Rubin, Elise and Florina.

## 1. Introduction

Apple tree (*Malus domestica*) is most frequently grown fruit tree in the Poland. Most of the harvested apples are processed—they are mainly used for the production of apple juice concentrate (about 90%), fresh juices, smoothies, droughts and alcoholic beverages [1]. The quality of alcoholic beverages is closely dependent on the quality of raw materials, yeast applied for fermentation and fermentation conditions. Fruits designated for processing should reach suitable maturity. The aroma of overripe fruits may be characterized by non-specific aroma notes associated with the development of epiphytic microbiota, while unripe fruit contain less sugar and are not suitable for the production of high-quality beverages [2]. Apart from compounds derived from raw material, volatiles are also formed in biologic, enzymatic and chemical processes throughout whole ethanol fermentation. Esters, aldehydes, higher alcohols, organic acids and terpenes are the most important aroma compounds of alcoholic beverages [3,4].

Esters are derived from raw materials, produced by yeast during fermentation in the reaction between alcohols and acetyl-CoA catalyzed by acetyltransferase, while some may be formed by transesterification as well. The process of ester formation is influenced by many factors, including fermentation temperature, pH, nitrogen level, microbiota present during fermentation and factors stimulating microbial growth [3].

Terpenes is a large group of aliphatic long-chain hydrocarbons, derived from active isoprene (isopentenyl diphosphate) or its isomer—dimethylallyl pyrophosphate. They are formed by combining two or more 5-carbon-atoms molecules leading to the formation of C10 monoterpenes, C15 sesquiterpenes or C20 diterpenes. There are various types of oxygenated derivatives of terpene hydrocarbons including alcohols (citronellol and linalool), aldehydes (citral and citronellal), ketones (mircenon and *o*-cymenon), esters or oxides [3].

Higher alcohols constitute between 0.1% to 0.7% of the quantity of produced ethanol. According to the Ehrlich theory, fusel alcohols are generated by decarboxylation and deamination of corresponding amino acids, for example, leucine is converted to 3-methyl-1-butanol, isoleucine to 2-methyl-1-butanol and valine to isobutanol [3,5].

Most data regarding volatile profile and sensory analysis of fruit brandies regards the determination of qualitative and quantitative profiles of volatile compounds, but it does not concern the impact of particular cultivars on such chemical substances in alcoholic beverages [6,7,8]. It seems that in the case of brandies produced from various cultivars of plums [9], cherries and pears [10] the selection of certain cultivars had the major impact on the profile of volatile compounds, and it was related to highest results obtained in sensory analysis. There are relatively few studies on apple brandies regarding the impact of apple cultivar used for fermentation on detailed profile of volatile compounds of apple distillates. Moreover, dessert apples are increasingly used to produce apple brandies. Therefore, in our experiments we used cultivars of dessert apple most commonly grown not only in Poland, but also in many other countries. The aim of the study was to determine the impact of dessert apple cultivar used for fermentation on the composition and concentration of volatiles in apple spirits. The results of our research will enrich the knowledge about the effect of the fruit cultivar on the quality of brandies—and also enable producers to choose a cultivar of apples to produce beverages with characteristic flavor.

## 2. Materials and Methods

### 2.1. Fermentation

Apple musts used for fermentation were obtained from ten different apple cultivars (Elise, Rubin, Topaz, Golden delicious, Szampion, Gloster, Pinova, Florina, Idared and Jonagored). Fruits were harvested in orchards in Garlica Murowana (50.1500° N, 19.9333° E, Małopolska district, Poland). Apples were washed, crushed, pressed and divided into 2 kg aliquots in 3 L sterile glass flasks. Musts were supplemented with (NH_4_)_2_HPO_4_ (0.2 g/kg raw fruit) and inoculated (0.3 g dry weight/L of must) with Ethanol Red (*Saccharomyces cerevisiae*) yeast strain (Starowar, Warsaw, Poland). Alcoholic fermentation was carried out for 30 days at 20 °C. Weight loss associated with the liberation of carbon dioxide was measured daily.

### 2.2. Distillation

First, fermented musts were distilled till the ethanol concentration in the collected distillate was lower than 0.5% (*w*/*v*). Then collected distillate was distilled as well and final ethanol concentration was ranging between 11.6–20.2% (*v*/*v*) of ethanol.

Then, the distillate was distilled again using a glass column (40 cm) filled up to 60% with Raschig rings and three fractions were collected: the heads (2% of the distillate), the heart fraction (83%) and the tails (15%). Final ethanol concentrations in apple brandies was approximately 65% (*v*/*v*), in head fraction 80% (*v*/*v*) and in tail fraction 20% (*v*/*v*), respectively. In order to avoid the loss of volatiles all fractions were kept at 4 °C in sealed flasks until further analysis. In the current study, we only presented results for heart fraction.

### 2.3. Analysis of Oenological Parameters

The ethanol content, total extract and sugar-free extract were determined using officially approved methods [11]. Titratable acidity (TA) was determined using Mettler DL 25 titrator (Greifensee, Switzerland). It was calculated from the volume of 0.1 M NaOH used for titration and expressed as gram of malic acid per liter. Fermentation efficiency (%) was calculated based on the relationship between sugar loss and ethanol produced following the fermentation stoichiometry, where 0.511 g or 0.538 g ethyl alcohol is obtained from 1 g of reducing sugars or sucrose, respectively. Free amino nitrogen (FAN) was determined with the ninhydrin method. The absorbance of samples was measured at a wavelength λ = 575 nm [12].

### 2.4. Determination of Sugar Content by High Performance Liquid Chromatography

Samples of apple musts before and after fermentation were centrifuged (MPW-65R, MPW Med. Instruments, Warszawa, Poland) at 14,000× *g*/5 min and fresh musts were diluted. Fermented musts were evaporated (Rotavapor R-220 SE, Buchi AG, Flawil, Switzerland) prior to analysis. Before injecting samples, we filtered them through syringe filters (0.45 µm pore density, Sartorius AG, Getinge, Germany). The analysis of sugar profile was carried out by high performance liquid chromatography (HPLC) method using Shimadzu apparatus (Kyoto, Japan) NEXERA XR equipped with the refractometer detector RF-20A. Separation was performed on the Asahipak NH2P-50, 4.6 × 250 mm Shodex column (Showa Denko America, Munich, Germany) thermostated at 30 °C. An aqueous solution of acetonitrile (70%) was the mobile phase and isocratic program elution (0.8 mL/min) lasted 16 min. Standard curves were prepared for the following substances: glucose, fructose, sucrose and glycerol. To validate the method we measured the concentration of mentioned substances in ten apple musts before and after fermentation and then we added known quantities (5, 10 and 20 g/L) of sugars or glycerol to those musts and carried out measurements again. We carried out that analysis in three replicated and we confirmed that added quantities were detected. Considering low detection limits (LOD) and low quantity limits (LOQ) (Table S1, Supplementary Materials) we confirmed that applied method was suitable for testing musts before and after fermentation. Moreover, R2 values indicated very high linearity within tested range of used standards.

### 2.5. Volatile Compounds Analysis by Gas Chromatography–Flame Ionization Detector and Solid Phase Microextraction-Gas Chromatography-Mass Spectrometry

Analysis of selected volatile compounds was carried out using gas chromatography as described by Satora and Tuszyński (2005) [13]. Gas chromatography–flame ionization detector (GC-FID) analysis was carried out on the Hewlett Packard 5890 Series II chromatograph system. Tested components were separated on the HP-INNOWAX capillary column (crosslinked polyethylene glycol stationary phase; 30 m × 0.53 mm ID with 1.0 μm film thickness, Agilent, Santa Clara, CA, USA). the temperature of detector and injector was 250 °C and the column was heated using the following temperature program: 35 °C for five minutes at increments of 5 °C/min to 110 °C, then 40 °C/min to 220 °C and maintained at constant temperature for three minutes. The carrier gas was helium at a 20.0 mL/min flow. Hydrogen flow speed was 33.0 mL/min, and that of air was 400 mL/min. Qualitative and quantitative identification of volatile substances (Sigma-Aldrich, Saint Louis, MO, USA) was based on the comparison of retention times and peak surface area read from sample and standard chromatograms and verified against results obtained for the internal standards (anethole, ethyl nonanoate and 4-methylo-2-pentanol). Concentrations of volatile components were recalculated based on 100% (*v*/*v*) ethanol and were expressed as mg/L.

In solid phase microextraction-gas chromatography-mass spectrometry (SPME-GC-MS) method, 2 mL of saturated saline with an internal standard solution (5 mg/L 4-methyl-2-pentanol and 0.05 mg/L ethyl nonanoate, Sigma-Aldrich) and 0.05 mL of spirit was added into 10 mL vials. The SPME device (Supelco, Inc., Bellefonte, PA, USA) coated with PDMS (polydimethylsiloxane), 100 μm fiber was first conditioned by inserting it into the GC injector port at 250 °C for 1 h. For sampling, the fiber was inserted into the headspace under stirring (300 rpm) for 30 min at 60 °C. Subsequently, the SPME device was introduced into the injector port of the Agilent Technologies 7890B chromatograph system equipped with LECO Pegasus HT, high throughput TOF-MS (time-of-flight mass spectrometry), and was kept in the inlet for 3 min. The SPME process was automated using the GERSTEL MultiPurpose Sampler (MPS, GERSTEL Inc., Linthicum, WA, USA).

Analyzed compounds were separated on a Rtx-1ms capillary column (Crossbond 100% dimethyl polysiloxane, 30 m × 0.53 mm × 0.5 μm). The detector temperature was 250 °C, and the column was heated using the following temperature program: 40 °C for three minutes at an increment of 8 °C/min to 230 °C, then maintained at constant temperature for 9 min. Carrier: helium at 1.0 mL/min constant flow. Electron impact mass spectrometry (EIMS) electron energy 70 eV; ion source temperature and connection parts: 250 °C. Analyte transfer was performed in splitless mode; the mass spectrometer-detector (MSD) was set to scan mode from *m/z* = 40 to *m/z* = 400.

Compounds were identified using mass spectral libraries and linear retention indices, calculated from a series of *n*-alkanes from C_6_ to C_30_. The quantity of volatiles was determined semi-quantitatively by measuring the relative peak area of each identified compound, according to the NIST (National Institute of Standards and Technology) database, in relation to that of the internal standard.

### 2.6. Sensory Analysis

Sensory analysis of apple brandies was based on aroma and included eight sensory descriptors (fruity, sweet, grassy, floral, smoked, citrus, pungent, yeast) rated in 5-point hedonistic scale in quantitative descriptive analysis (QDA). Panelists were selected among scientific staff working in the faculty of food technology and human nutrition who previously graduated from that faculty and obtained extensive course of sensory analysis as a part of their curriculum. Aroma evaluation was determined using a set of standards provided to panelists prior to analysis [14]. First, panelists received standards of various aromas determine whether they were able to recognize each of them. Then they received the same standards, but at various concentrations. Only those who passed those two stages were selected as panelists. Apple brandies (diluted to 40% vol. EtOH) were subjected to sensory assessment by the panel comprising of 10 panelists. Samples were coded and provided to panelists in randomized order. Results were subjected to one-way analysis of variance (ANOVA) an than Pearson test was carried out for each descriptor (Table S2, Supplementary Materials).

### 2.7. Statistical Analysis

All experiments were performed at least in five replicates and results were presented as arithmetic means ± standard deviation. Statistical analysis was carried out in the R 3.5.0 (Vienna, Austria) program. The ANOVA was carried out using linear model (lm) function and Tukey’s test was done using honest significant difference (HSD) test function in agricolae’package.

## 3. Results and Discussion

### 3.1. Selected Chemical Parameters of Fresh and Fermented Apple Musts

Fresh, matured apples contain about 10–13% total sugars, among which fructose dominates [15]. Fructose was dominant in sugar profile (over 50% of total sugar) in analyzed apple musts (Table 1). The average concentration of glucose was much lower (9.7–25.5 g/L). There are various organic acids present in apple fruit, including malic, citric, succinic, quinic and galacturonic acids. These acids occur as free molecules or they are bound to other compounds in must. Their concentration has a significant impact on taste, pH, fermentation and beverage stability [16]. The acidity of certain musts used in the experiment were slightly lower than those found in the studies by Tarko et al. (2018) [17], nonetheless, the acidity increased after fermentation and ranged from 3.73 to 7.32 g/L. According to legal regulations in Poland [18], total acidity in fermented fruit beverages should range from 3.5 g to 7 g of malic acid per liter.

The nitrogen fraction of apples includes amino acids such as asparagine, glutamine, aspartic acid, glutamic acid and serine, which dominate the profile of amino acids (86% to 95% of total amino acids) and can be easily assimilated by yeast. The content of nitrogen in apples depends on the age of orchards, area of cultivation and the type and amount of used fertilizers. Fruits harvested from trees growing on intensely fertilized soils can contain up to five times higher concentrations of nitrogen compounds than on average [19]. Fresh musts analyzed in our studies contained relatively low amounts of nitrogen compounds (13.7–61.7 mg/L), therefore, the supplementation with ammonium hydrogen phosphate was necessary.

**Figure 1:**
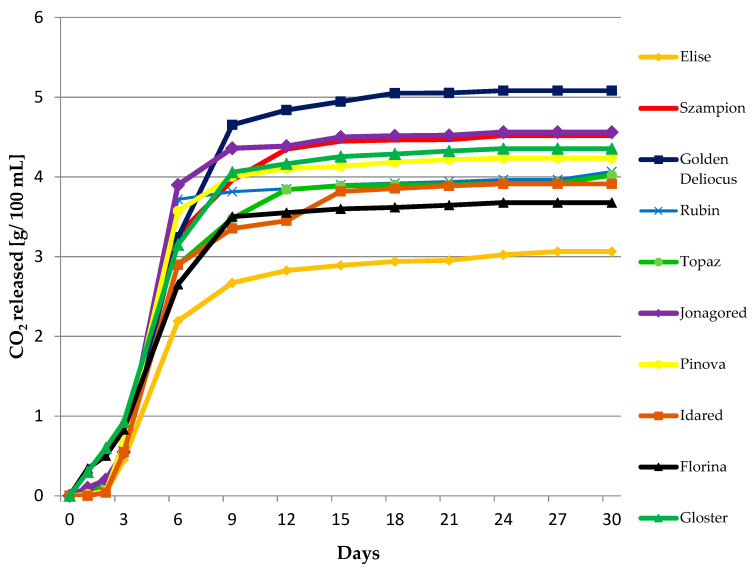
Fermentation dynamics of apple musts, *n* = 3, STD < 5%.

Musts obtained from different apple cultivars demonstrated variable fermentation dynamics (Figure 1). The turbulent fermentation phase began first, in Gloster and Florina musts, while in other samples it was observed about a day later. Similar phenomenon was observed by Satora et al. (2008) [20], who stated that musts obtained from Gloster cultivar fermented earlier than musts obtained from other cultivars. The optimal fermentation rate was noted for Golden delicious musts—the turbulent fermentation lasted about eight days and highest final weight losses were recorded (about 5 g/100 mL) (Figure 1).

The fermentation efficiency ranged from 61.3% (Idared) to 94.7% (Gloster). In all samples, over 80% of reducing sugars was used during fermentation, with glucose being utilized in largest quantities (residual glucose from 0.03 to 0.28 g/L). During fermentation *S. cerevisiae* yeast initially uses glucose available in the medium, followed by other simple sugars and disaccharides [17]. The quantity of ethyl alcohol produced during the fermentation is mainly determined by the level of fermentable sugars. In apple musts not supplemented with sucrose, generally around 5% of ethanol is produced [10]. Ethanol content in fermented samples varied from 4.1 (Idared) to 6.3% vol. ethanol (Topaz) (Table 2). Similar or higher concentrations of ethanol in apple wines obtained from Rubin, Elise and Topaz fermented using Ethanol Red yeast were demonstrated by Tarko et al. (2018) [17].

### 3.2. Volatile Compounds

Qualitative and quantitative profile of terpenes in apple brandies has not been studied in detail so far. The presence of eugenol, chavicol and isoeugenol in apple fermented beverages was already shown [21]. In case of analyzed samples, eugenol was present in high concentrations (2.33–8.28 mg/L 100°). Significantly lower content of this compound (below 1 mg/L) was detected in apple spirits analyzed by Rodríguez-Madrera and Mangas Alonso (2010) [22] and in plum brandies analyzed by Satora et al. (2016) [9] (below 0.2 mg/L). Higher content of eugenol in apple spirits (6 mg/L) was demonstrated by Coldea et al. (2011) [6]. Analyzed brandies contained also high concentration of isoeugenol and β-ionone. Terpinen-4-ol was present in similar concentrations in all samples (about 1 mg/L 100°) (Table 3). This compound was characteristic for apple spirits analyzed by Bajer et al. (2017) [7]. The presence of β-citronellol distinguished four apple brandies and its highest content was noted in spirits obtained from fermented musts of Rubin and Szampion cultivars. Linalool oxide was present in all analyzed spirits (0.51–0.77 mg/L 100°) and its concentration was higher than in grape (0.29 mg/L 100°) and plum spirits (0.21 mg/L 100°) [8]. Myrcene and limonene were characteristic for apple brandies obtained from Topaz and Elise cultivars. Limonene is not commonly present in apple brandies. Its presence was also found in Earligold apple [23]. Gas chromatography- mass spectrometry method enables the detection of some other components, e.g., nerolidol, α-phellandrene, o-cymene, α-terpineol and β-damascenone (Table 4). Nerolidol with characteristic rose/keiskei/apple blossom flavor was present in highest concentration in all analyzed samples. This compound is also the major compound responsible for the aroma of grape spirits [24].

Ethyl acetate is the most abundant ester, generally exceeding 80% of all esters in fruit spirits [25]. In analyzed apple spirits, ethyl acetate was also predominant ester reaching over 25% of all volatile esters (Table 3 and Table 4). The highest amounts of these compounds were detected in apple brandy obtained from Topaz cultivar (199 mg/L 100°) and the lowest from Jonagored apples (105 mg/L 100°). Much higher content of ethyl acetate (198.2–744.2 mg/L) in spirits obtained from distilled ciders was shown by Rodríguez-Madrera and Suárez Valles (2007) [26] which could be related to the involvement of particular microorganisms in fermentation. Acetate esters of higher alcohols and ethyl esters of fatty acids are significant volatile compounds in spirits. Peng et al. (2009) [27] reported that one of the key aroma components in ciders is isoamyl acetate. Samples analyzed in our research contained relatively high concentrations of that compound (73–145 mg/L 100°, Table 3). Analyzed apple brandies contained below 2 mg of ethyl caproate per liter of 100° (Table 3). Similar or higher concentrations were detected in apple spirits (1.92–12.82 mg/L) by Rodríguez-Madrera and Suárez Valles (2007) [26]. Ethyl caprate was detected almost at the same level in all apple spirits (about 8 mg/L 100°). The presence of this compound in apple brandies was confirmed by Bajer et al. (2017) [7]. Similarly, diethyl succinate was detected at similar levels in all analyzed samples. According to other researches, diethyl succinate commonly occurs in alcoholic beverages, e.g., plum brandies (2 mg/100 mL 100°), mirabelle brandies (3.5 mg/100 mL 100°), Scotch whiskies (0.3 mg/100 mL 100°), cognac and Armagnac (1 mg/100 mL 100°) [28]. Ethyl laurate was present at relatively high concentrations in analyzed samples (about 4 mg/L 100°). The content of ethyl laurate was significantly lower (about 1 mg/L 100°) [29] in ten apple spirits purchased from local markets in Asturias (Spain) than its concentration demonstrated in the current study. This ester is characterized by fruity and waxy aroma and could be a characteristic component of apple spirits either because its concentration was rather high, and it was present in all samples. Methyl valerate was detected only in one sample (spirits obtained from Topaz cultivar). Ethyl caprylate has a fruity aroma and it is found in many species of fruits, e.g., apple, apricot, orange, grapefruit, guava, pineapple, passion fruit and mango [30]. This compound was present at highest concentrations in brandies obtained from Topaz, Szampion and Idared cultivars. Rodríguez-Madrera and Suárez Valles (2007) demonstrated lower content of this compound in apple spirits (3.03–15.36 mg/L) [26]. Gas chromatography-mass spectrometry method enables the detection of more than 30 other esters (Table 4). As in the case of terpenes, the spirits obtained from Gloster contained the smallest concentrations of esters. Some of esters were characteristic for spirits obtained from specific cultivar, for example benzyl acetate for Elise spirits and ethyl *trans*-4-decanoate for Jonagored. Benzoates (ethyl benzoate and benzyl benzoate) were a relatively small group of esters found in studied apple spirits present in low concentrations (Table 4). Benzoates in higher concentrations provide characteristic wintergreen-like flavor [31].

Amyl alcohols are quantitatively the largest group of higher alcohols in analyzed distillates. They are responsible for the flavor of alcoholic beverages, and the quality of those drinks depends on their concentration [3]. The content of those compounds in analyzed samples ranged from 867 mg/L (Idared) to 1802 mg/L 100° (Szampion). Spaho (2017) [25] confirmed that the largest share in the group of higher alcohols was assigned to amylic alcohol. The highest propanol concentration was noted in spirits obtained from Rubin cultivar (201 mg/L 100°). Rodríguez-Madrera and Suárez Valles (2007) [26] demonstrated that the concentration of that compound in spirits made from ciders ranged from 92.25 to 400.53 mg/L 100°. Butanol and isobutanol were present in all analyzed spirits. In the current study, concentration of isobutanol exceeded 230 mg/L 100° and amounts of butanol ranged from 8.1 to 34.0 mg/L 100°. These values were lower than those reported by Coldea et al. (2011) [6] who analyzed apple brandies. In the case of hexanol, its concentration ranged from 78.3 to 91.5 mg/L 100°. Hexanol is responsible for the grassy scent in distillates, however, when the concentration of that compound exceeds 100 mg/L 100°, it deteriorates sensory properties of spirits [25].

The maximum acceptable methanol content in apple spirits distributed in the European Union is 12 g/L 100° vol. alcohol [32]. In the current study, the concentration of that substance in analyzed brandies was much lower (Table 3). In apple spirits tested by Croitoru et al. (2013) [33] methanol content reached 0.5% and the highest amount of this compound was characteristic to spirits obtained from apple-plum musts (1%). Spirits obtained after cider distillation demonstrated significantly lower methanol content—from 203 to 679 mg/L [25]. Boiling temperature of methanol is only 64.7 °C so most that compound is transferred to heads fraction.

The aroma of studied apple spirits was also evaluated according to other detected components such as aldehydes and ketones (Table 3 and Table 4). Acetaldehyde, which dominates in aldehyde profile (about 90%) in brandies [25], was present in all analyzed samples in concentration range from 92.4 to 226 mg/L 100°. Similar concentration of this compound was determined in different alcoholic beverages, e.g., wine distillates (37–111 mg/L 100°), brandies (126–595 mg/L 100°), Kirsch (110–170 mg/L 100°), apple brandies (140 mg/L 100°) and plum brandies (120 mg/L 100°) [28]. In low concentration aroma of acetaldehyde resembles cherry, hazelnuts and overripe apples, however, when the concentration of that compound exceeds 1.2 g/L 100°, it deteriorates sensory properties of spirits. Other carbonyl compounds were present in relatively high concentrations and most of them were present in all analyzed samples (e.g., furfural, benzaldehyde and 6-methyl-5-hepten-2-one). Several substances from that group were characteristic in spirits from particular apple cultivars: 1,1-diethoxy-propane from Topaz and Idared cultivars; hexanal from Elise, Golden delicious and Topaz; and benzothiazole from Elise, Szampion, Topaz and Idared.

### 3.3. Sensory Analysis

**Figure 2:**
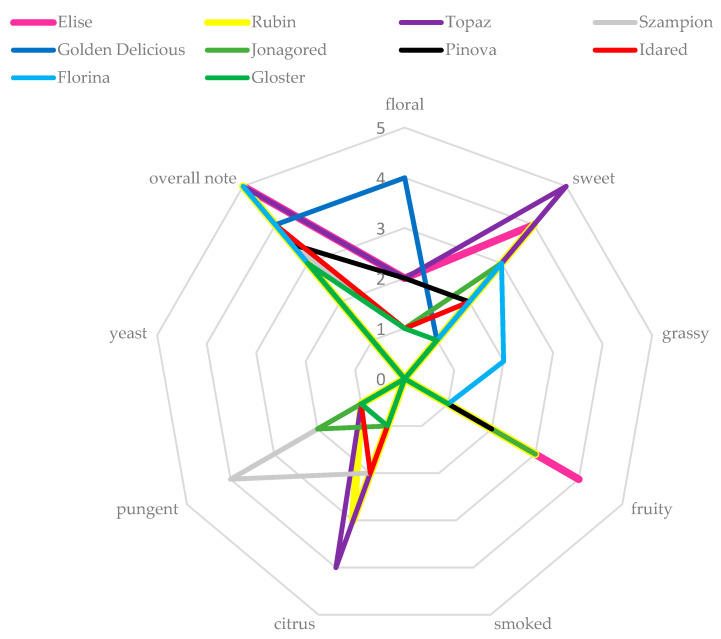
Characteristic aroma traits of apple spirits obtained from various apple cultivars fermented by Ethanol Red *(Saccharomyces cerevisiae)* yeast.

All analyzed samples were described as clear and obtained maximal notes for that parameter. Among 10 analyzed apple spirits the highest scores for the parameter “overall note” obtained—Eliza, Rubin, Topaz and Florina. Brandies obtained from Topaz cultivar characterized sweet (5 points) and citrus (4 pts) aroma (Figure 2), which could be associated with the most diverse profile of volatile compounds, especially the highest concentration of most of terpenes, e.g., α-phellandrene, *o*-cymene, α-terpineol (SPME-GC-MS) and citral, myrcene (GC-FID). Only the aroma of spirits obtained from Florina cultivar was described as grassy (2 out of 5 pts) which could be related to the highest concentration of hexanol (grassy-green notes) and linalool oxide with characteristic tea tree aroma [4]. The aroma of spirits obtained from Golden delicious cultivar was described as floral (4 out of 5 pts) which could be related to the presence of isoeugenol (clove aroma), eugenol (clove aroma) and β-ionone with characteristic violet aroma [4]. Pungent aroma recognized in spirits obtained from Szampion cultivar could be attributed to the highest concentration of fusel alcohols and eugenol; the latter compound is responsible for clove aroma which could result in pungent and burning flavor [30]. Spirits made from Gloster cultivar which received lowest scores in sensory evaluation (overall note 3 out of 5 pts) did not contain any of analyzed terpenes, i.e., limonene, (-)-β-citronellol, myrcene and none of acetate esters analyzed with SPME-GC-MS. Pearson test indicated strong positive correlations between some descriptors (floral, sweet, fruity or citrus) and overall note. Moreover, there were negative correlations between pungent descriptor and overall note.

## 4. Conclusions

Our research proved that dessert apples could be used for the production of apple brandies and confirmed the hypothesis that the fruit cultivar significantly influences volatile profile and sensory characteristics of obtained spirit. Of the ten analyzed apple spirits, those obtained from Topaz, Rubin and Elise cultivars demonstrated the most diverse profile of volatile compounds. Moreover, their oenological parameters that are the most important in the production of alcoholic beverages were the most favorable. Brandies obtained from Gloster contained the smallest concentrations of esters and terpenes. Characteristic volatiles for brandies obtained from Topaz were limonene, myrcene, methyl valerate and 1,1-diethoxy-propane; Rubin—β-citronellol and isopropyl acetate; Elise—limonene, myrcene, benzyl acetate and isopropyl acetate; Szampion—β-citronellol; Idared—1,1-diethoxy-propane and Jonagored- ethyl *trans*-4-decanoate. Eugenol, β-ionone, β-damascenone and nerolidol were present at highest concentrations of terpenes in most of analyzed alcoholic beverages. Ethyl acetate was the most characteristic ester occurring in apple brandies and it dominated ester profile (about 30% of total esters). Results of the sensory analysis showed that the highest scores brandies were obtained from Topaz, Rubin, Elise and Florina. Those brandies demonstrated pleasant, sweet, fruity, citrus and alcoholic aroma. The results of this research will enrich the knowledge about the effect of the fruit cultivar on the quality of fruit brandies, and also enable producers to choose a cultivar of apples to produce beverages with new, unique characteristics. Topaz, Rubin and Elise cultivars were used for further unpublished studies on the production of apple brandies.

## Figures and Tables

**Table 1:** Sugar composition of fresh and fermented musts obtained from various apple cultivars.

Apple Cultivars	Before Fermentation Processes					After Fermentation Processes				
	Glycerol	Fructose	Glucose	Sucrose		Glycerol	Fructose	Glucose		Sucrose
	(g/L)									
Elise	0.00 ± 0.00	56.41e ± 1.81	9.71e ± 0.71		14.71d ± 0.62	4.74ab ± 0.24	0.53ab ± 0.36	0.25abc ± 0.02	0.16a ± 0.13	
Rubin	0.00 ± 0.00	61.12a ± 0.93	25.52a ± 0.33		13.42cd ± 0.44	5.57ab ± 1.06	0.52ab ± 0.19	0.08bc ± 0.04	0.08a ± 0.03	
Topaz	0.00 ± 0.00	70.31c ± 0.81	24.94b ± 0.61		10.52d ± 1.11	5.77ab ± 0.72	0.25ab ± 0.13	0.12abc ± 0.06	0.13a ± 0.04	
Szampion	0.00 ± 0.00	65.41c ± 0.21	14.71d ± 0.92		17.62b ± 0.53	4.94ab ± 0.38	0.86a ± 0.24	0.20ab ± 0.02	0.12a ± 0.11	
Golden delicious	0.00 ± 0.00	47.61bc ± 0.11	17.43c ± 0.83		24.31a ± 1.82	7.26a ± 1.95	0.31ab ± 0.25	0.21a ± 0.13	0.25a ± 0.17	
Jonagored	0.00 ± 0.00	54.73d ± 0.91	20.42b ± 0.61		15.91bc ± 0.72	3.88b ± 0.39	0.42ab ± 0.11	0.03c ± 0.05	0.11a ± 0.08	
Pinova	0.00 ± 0.00	56.42c ± 0.51	21.93b ± 1.32		18.81b ± 0.75	4.28ab ± 0.91	0.28ab ± 0.17	0.28abc ± 0.16	0.10a ± 0.07	
Idared	0.00 ± 0.00	60.31ab ± 0.01	15.61cd ± 0.91		23.21a ± 0.02	6.27ab ± 1.26	0.17b ± 0.19	0.04c ± 0.11	0.03a ± 0.03	
Florina	0.00 ± 0.00	51.92d ± 1.61	14.64d ± 1.42		17.21b ± 2.71	4.32ab ± 1.73	0.67ab ± 0.25	0.03c ± 0.04	0.06a ± 0.03	
Gloster	0.00 ± 0.00	50.01f ± 0.41	16.91f ± 0.21		16.11bc ± 1.41	4.59ab ± 1.11	0.25ab ± 0.13	0.03c ± 0.04	0.06a ± 0.03	
Significance	ns	***	***		***	*	*	**	ns	

Same letters next to mean values within columns indicate the lack of statistically significant differences at *p* < 0.05; *n* = 3; *ns*—not significant; 0.001= ***; 0.01 = **; 0.05 = *.

**Table 2:** Selected chemical parameters of fresh and fermented musts obtained from various apple cultivars.

Apple Cultivars	Before Fermentation Processes				After Fermentation Processes					
	Total Extract	Sugar-Free Extract	Titratable Acidity	Free Ammonia Nitrogen (Fan)	Total Extract	Sugar-Free Extract	Titratable Acidity	Free Ammonia Nitrogen (fan)	Ethanol Content	Fermentation Efficiency
	(g/L)			(mg/L)	(g/L)			(mg/L)	(% vol.)	(%)
Elise	115.0a ± 2.0	24.2b ± 1.4	4.86b ± 0.08	53.1b ± 1.0	9.0e ± 1.0	8.1d ± 0.8	4.01cd ± 0.33	24.7ab ± 1.7	5.1bc ± 0.2	93.3b ± 0.6
Rubin	115.0a ± 2.0	15.0c ± 0.8	4.22c ± 0.18	43.3c ± 0.9	13.0de ± 1.5	12.3b ± 1.5	5.02bc ± 0.67	26.4a ± 8.5	6.2a ± 0.2	91.7c ± 0.6
Topaz	120.0a ± 1.0	14.3d ± 0.3	5.51a ± 0.36	31.9e ± 0.1	21.0ab ± 1.0	20.5a ± 1.3	7.32a ± 0.18	13.2bcd ± 1.9	6.3a ± 0.3	88.2d ± 0.5
Szampion	112.0a ± 3.0	14.3d ± 0.5	3.39def ± 0.13	45.4c ± 0.7	14.0cd ± 2.0	12.5b ± 0.7	3.73d ± 0.47	16.2abc ± 1.8	6.2a ± 0.1	93.8b ± 0.4
Golden delicious	119.0a ± 1.0	29.7a ± 2.1	3.28ef ± 0.12	16.6f ± 1.7	21.0ab ± 1.5	19.9a ± 1.2	4.91cd ± 0.44	10.9cde ± 2.9	4.2d ± 0.3	69.7f ± 0.3
Jonagored	104.0b ± 1.5	13.0de ± 1.3	3.32def ± 0.19	61.7a ± 0.7	19.0bc ± 2.0	18.4a ± 1.7	5.01ab ± 0.68	16.3abc ± 3.56	5.7b ± 0.2	92.7bc ± 0.5
Pinova	114.0a ± 2.0	16.9c ± 1.9	2.94f ± 0.05	28.1e ± 0.6	11.0de ± 2.0	10.3c ± 0.7	5.32bc ± 0.74	8.3de ± 5.96	4.9c ± 0.2	74.7e ± 0.3
Idared	104.0b ± 1.5	4.9f ± 0.1	3.73cd ± 0.05	13.7f ± 0.6	15.0cd ± 1.0	14.8b ± 1.8	5.93ab ± 0.68	13.3bcd ± 0.6	4.1bc ± 0.5	61.3 g ± 0.4
Florina	104.0b ± 2.0	20.3b ± 2.5	2.11 g ± 0.17	29.3e ± 1.5	6.0f ± 2.5	5.2e ± 0.7	4.72bc ± 0.77	22.5abc ± 2.7	5.2bc ± 0.2	91.8c ± 0.7
Gloster	104.0b ± 1.5	21.0b ± 0.4	3.69cde ± 0.06	41.8d ± 2.7	18.0ab ± 1.5	17.7a ± 1.3	5.64bc ± 0.72	18.2abc ± 6.1	5.3c ± 0.3	94.7a ± 0.4
Significance	***	***	***	***	***	***	***	***	***	***

Same letters next to mean values within columns indicate the lack of statistically significant differences at *p* < 0.05; *n* = 3; *ns*—not significant; 0.001= ***.

**Table 3:** Volatile compounds of brandies obtained by distillation of fermented musts from different apple cultivars by gas chromatography–flame ionization detector (GC-FID).

Terpenes	Elise	Rubin	Topaz	Szampion	Golden Delicious	Jonagored	Pinova	Idared	Florina	Gloster	Significance
	**(mg/L 100°)**										
Limonene	0.39a	0.00c	0.21b	0.00c	0.00c	0.00c	0.00c	0.00c	0.00c	0.00c	***
Linalool oxide	0.51b	0.58b	0.75a	0.55b	0.53b	0.75a	0.65ab	0.75a	0.77a	0.76a	***
Linalool	0.35b	0.35b	0.37a	0.33b	0.35b	0.36ab	0.37a	0.36ab	0.35ab	0.34b	***
(+)-terpinen-4-ol	0.94a	0.94a	0.93a	0.92a	0.90a	0.94a	0.89a	0.90a	0.92a	0.88a	ns
Citral	0.28a	0.26a	0.31a	0.27a	0.29a	0.25ab	0.30a	0.28a	0.24ab	0.23b	***
Geraniol	0.18a	0.19a	0.20a	0.19a	0.17a	0.21a	0.24a	0.20a	0.21a	0.17a	ns
β-ionone	2.86d	3.41c	6.41a	5.01b	5.71ab	3.96c	6.48a	5.90ab	3.21c	4.72b	**
Isoeugenol	1.60bc	1.88ab	1.19 g	1.67b	1.93a	1.42d	1.49cd	1.35e	1.32ef	1.27f	**
Methyl eugenol	0.18b	0.33a	0.17b	0.31a	0.00c	0.33a	0.16b	0.00c	0.00c	0.28a	***
(-)-ß-citronellol	0.00d	0.38ab	0.00d	0.37ab	0.00d	0.19bc	0.00d	0.00d	0.15c	0.00d	***
Eugenol	6.46cd	7.55c	3.27ef	8.28a	7.58ab	6.24cd	5.81e	2.33 g	5.57e	2.97f	**
Guaiacol	0.26d	0.42cd	2.77ab	2.31ab	2.49ab	2.81a	2.02aC	2.67ab	2.56ab	0.62cd	***
Myrcene	0.08a	0.00b	0.11a	0.00b	0.00b	0.00b	0.00b	0.00b	0.00b	0.00b	***
**Esters**											
Isoamyl acetate	104ab	86bc	145a	84bc	88bc	79bc	73bc	96abc	79bc	90bc	**
Ethyl caproate	1.84a	1.89a	1.84a	1.87a	1.82a	1.88a	1.78a	1.84a	1.85a	1.84a	ns
Ethyl caprate	7.79a	7.87a	8.15a	7.88a	7.73a	7.94a	7.83a	7.79a	7.73a	7.66a	ns
Diethyl succinate	5.64b	5.11cd	5.18cd	5.97a	5.27c	5.17cd	5.65b	5.69b	5.21cd	5.23cd	***
Ethyl laurate	4.50bc	4.44c	4.53bc	4.47c	4.58bc	4.43c	4.49c	4.81a	4.80a	4.44c	***
Methyl anthranilate	70.6a	63.7a	68.8a	74.3a	71.3a	62.7a	68.6a	69.0a	61.4a	69.3a	ns
Methyl valerate	0.00b	0.00b	19.9a	0.00b	0.00b	0.00b	0.00b	0.00b	0.00b	0.00b	***
Isopropyl acetate	0.15a	0.08b	0.00c	0.00c	0.00c	0.00c	0.00c	0.00c	0.00c	0.00c	***
Ethyl caprylate	18.7b	12.7c	22.7a	20.8ab	9.7d	12.9c	10.7d	22.7a	12.8c	8.8d	***
2-phenylethyl acetate	112de	116c	117c	118bc	117c	115cd	117c	116c	112cd	120a	***
Ethyl acetate	123bc	192b	199a	192b	133bc	105c	137bc	127bc	193b	191b	***
**Other Compounds**											
Acetaldehyde	156b	199ab	156b	92c	167b	174b	227a	178b	215ab	204ab	***
Methanol	8462b	5424d	7684c	4588e	9379a	8734b	9873a	8925b	8325bc	9236a	***
Propanol	162a	201a	197ab	114c	157b	199ab	171ab	113c	173b	111c	***
Butanol	332d	244e	690a	620b	656ab	321d	430c	696a	234e	267e	***
Isobutanol	10.2c	19b	15.8bc	12c	8.1c	19b	10c	18.1b	34.0a	26.3ab	***
Hexanol	83.7a	79.4a	78.3a	81.5a	82.1a	79.4a	80.9a	83.7a	91.5a	81.5a	***
Amyl alcohols	1576b	1151d	1194c	1802a	1217c	1153cd	1004d	867e	1381c	983e	***

Color determination from lowest (0%) to highest (100%) concentration of volatile compounds.










Same letters next to mean values within rows indicate the lack of statistically significant differences at *p* < 0.05; *n* = 3; *ns*—not significant, 0.001= ***; 0.01 = **.

**Table 4:** Aroma composition of apple spirits produced from different apple cultivars (solid-phase microextraction- gas chromatography-mass spectrometry (SPME-GC-MS) (µg/L 100°).

Acetate Esters	LRI ^2^	Elise	Rubin	Topaz	Szampion	Golden Delicious	Jonagored	Pinova	Idared	Florina	Gloster	Sig.
	(µg/L 100°)											
Isobutyl acetate	763	57.1a	28.0bc	18.8cd	45.6ab	0.0d	10.1cd	4.5d	0.0d	0.0d	0.0d	***
Butyl acetate	799	36.9b	32.3b	6.5bc	90.6a	0.0c	35.0b	37.5b	27.6bc	0.0c	0.0c	***
1-Butanol, 2-methyl-, acetate	879	63.2bc	125.4b	0.0c	307.0a	0.0c	0.0c	77.7bc	63.5bc	0.0c	0.0c	***
Hexyl acetate	1006	674.1bd	2042.1a	712.9bc	906.4b	0.0d	89.4cd	377.8bd	121.8cd	0.0d	0.0d	***
Benzyl acetate	1137	15.4a	0.0b	0.0b	0.0b	0.0b	0.0b	0.0b	0.0b	0.0b	0.0b	***
Octyl acetate	1196	68.0a	37.7ab	49.3ab	4.9b	0.0b	8.9b	5.8b	0.0b	0.0b	0.0b	***
Decyl acetate	1394	227.4a	83.8ab	107.5ab	0.0b	0.0b	8.6b	0.0b	0.0b	0.0b	0.0b	**
**Methyl and Ethyl Esters**												
Ethyl butyrate	789	20.2ab	55.9a	55.5a	38.8ab	0.0b	27.0ab	4.6b	16.1ab	0.0b	0.0b	***
Ethyl-2-methylbutyrate	841	0.0c	0.0c	2.7b	21.3a	0.0c	0.0c	11.7a	4.4b	0.0c	0.0c	***
Ethyl 2-hydroxy-4-methylvalerate	1060	20.0ad	10.0cd	31.7ab	34.5a	0.0d	17.0ad	6.7cd	17.2ad	11.0bd	23.3ac	**
Methyl octanoate	1108	14.3a	36.6a	5.5a	20.5a	0.0a	9.4a	20.6a	2.3a	0.0a	0.0a	ns
Ethyl phenyl acetate	1210	116.9a	226.4a	110.5a	787.3a	47.3a	282.6a	47.2a	106.1a	77.1a	306.5a	ns
Ethyl 4-methyloctanoate	1252	227.7a	27.0b	23.5b	516.3a	24.1b	0.0b	15.5b	2.3b	0.0b	0.0b	***
Ethyl *trans*-4-decanoate	1357	0.0b	0.0b	0.0b	0.0b	0.0b	15.5a	0.0b	0.0b	0.0b	0.0b	*
Ethyl 9-decenoate	1366	55.6a	78.8a	0.0a	73.5a	0.0a	7.1a	0.0a	69.6a	5.3a	60.4a	ns
Methyl dodecanoate	1507	291.0ac	632.7ab	99.2bc	681.1a	1.2c	217.2ac	36.6c	3.7c	36.6c	67.1c	***
Ethyl tetradecanoate	1790	1470b	4584ab	20,771a	11,734ab	16b	3246ab	111b	68b	360b	996b	**
Ethyl pentadecanoate	1880	34.8b	70.0b	346.3a	125.3ab	0.0b	84.5b	6.3b	0.0b	22.9b	34.9b	**
Methyl hexadecanoate	1927	45.2a	257.7a	223.3a	390.1a	0.0a	208.7a	48.2a	28.6a	128.1a	66.0a	*
Ethyl 9-hexadecenoate	1977	102.3b	282.0b	1121.1a	591.0ab	0.0b	232.9b	30.5b	17.4b	82.5b	123.6b	**
Ethyl hexadecanoate	1990	1878.5b	6249.2b	32,808.7a	16,294.2ab	95.1b	6127.5b	383.6b	248.7b	2018.8b	3939.4b	*
Methyl 9,12-octadecadienoate	2147	227.8b	666.3ab	322.8b	2088.1a	0.0b	873.8ab	58.0b	40.0b	438.2ab	468.8ab	*
Ethyl octadecanoate	2189	7.5a	7.1a	54.0a	70.9a	18.5a	21.5a	0.0a	3.1a	10.9a	29.8a	*
**Benzoates**												
Ethyl benzoate	1142	21.7a	24.3a	7.5a	23.54a	0.0a	0.0a	8.8a	34.1a	11.2a	36.3a	ns
Benzyl Benzoate	1750	49.7ab	62.5a	40.2abc	41.5abc	0.0c	39.9abc	9.1bc	15.9bc	46.5ab	26.9abc	**
**Other Esters**												
Hexyl butyrate	1174	2.8b	28.1a	6.7ab	0.0b	0.0b	7.7ab	3.6b	0.0b	0.0b	0.0b	*
Hexyl 2-methylbutanoate	1222	98.3b	57.8c	37.1c	363.6a	0.0d	235.0a	139.5a	20.0c	47.3c	0.0d	***
Isopentyl hexanoate	1238	44.6b	21.7b	49.0b	193.3a	0.0c	21.7b	6.8b	4.9b	3.1b	6.2b	***
Isobutyl octanoate	1341	41.8ab	17.0ab	30.3ab	65.2a	0.0b	22.6ab	9.5ab	0.0b	10.2ab	17.0ab	*
Hexyl hexanoate	1372	36.3a	63.9a	160.7a	40.9a	0.0a	5.7a	4.2a	0.0a	0.0a	0.0a	ns
á-Phenylethyl butanoate	1411	26.4ab	41.9ab	63.3a	36.5ab	0.0b	27.1ab	12.8b	20.5ab	4.7b	14.0b	**
3-methylbutyl octanoate	1445	1350.0a	1098.5ab	904.8abc	887.5abc	0.0c	438.2abc	68.4bc	33.5bc	60.5bc	216.1bc	**
2-methylbutyl octanoate	1449	176.9a	123.6ab	13.7b	73.4ab	0.0b	53.3ab	8.3b	0.0b	4.1b	22.7b	**
Propyl decanoate	1472	67.2a	26.6b	19.9b	21.1b	4.8b	17.7b	0.0a	0.0a	3.3a	5.3a	***
Dibutyl maleate	1505	0.0b	0.0b	133.7a	0.0b	0.0b	49.4ab	16.9b	31.1b	28.6b	37.9ab	**
2-phenylethyl hexanoate	1611	395.1c	1508.3ab	1040.9b	2827.8a	30.8c	1089.8b	605.0bc	2917.8a	195.2c	448.6c	**
Isoamyl decanoate	1641	3738b	8239ab	50,019a	17,682ab	0.0b	5378a	193b	119b	172b	839b	*
Isobutyl laureate	1753	29.5b	47.3b	436.1a	146.7ab	0.0b	45.2b	0.0b	0.0b	4.0b	13.8b	***
Hexyl decanoate	1784	78.7b	172.1ab	714.6a	249.1ab	42.7b	142.1b	86.1b	33.5b	45.4b	12.4b	**
2-phenylethyl octanoate	1820	487.0b	2092.5b	25,675.0a	1892.1b	0.0b	2668.8b	459.6b	2087.6b	496.2b	1312.5b	**
Isoamyl laureate	1844	56.5ab	42.1b	50.0b	155.4a	12.2b	46.7b	26.0b	30.5b	49.7b	107.9ab	***
**Alcohols**												
4-methyl-1-Pentanol	821	25.9de	121.1ac	177.5ab	179.5a	0.0c	79.7cd	104.7bc	182.9a	26.9de	115.7ac	***
3-methyl-1-Pentanol	825	485.6e	928.0ce	1863.9ab	1620.3bc	339.1e	800.7de	1460.2bd	2520.1a	407.8e	629.6e	***
3-Hexen-1-ol	845	87.7c	83.7c	600.2b	369.7bc	53.8c	209.6bc	0.0c	1710.1a	0.0c	1663.5a	***
1-Heptanol	954	75.4c	368.0b	269.2bc	679.1a	38.1c	49.5c	141.8bc	189.5bc	29.6c	129.4bc	***
1-Octanol	1070	246.0ac	518.9a	385.0ac	479.5ab	33.2bc	47.8bc	246.8ac	101.6ac	0.0b	27.3bc	**
Phenyl ethanol	1084	10,865b	15,788b	47,775ab	36,626ab	86,592a	13,934b	43,232ab	66,136ab	15,692b	19,896ab	***
1-Nonanol	1156	120.6bc	1049.8a	241.5ac	1027.1ab	89.7c	0.0c	138.0bc	153.4ac	119.5bc	122.7bc	**
1-Decanol	1272	78.7a	229.1a	215.1a	278.4a	99.9a	99.3a	70.9a	89.8a	52.9a	136.5a	ns
6,10-dimethyl-5,9-Undecadien-2-ol	1455	0.0c	0.0c	0.0c	0.0c	0.0c	0.0c	253.7ab	9.8c	335.9a	65.6bc	***
Phenol, 2,4-bis(1,1-dimethylethyl)-	1490	563.1b	1039.6a	238.5b	1065.9a	140.5b	766.9b	444.4b	456.4b	689.5b	478.5b	***
1-Tetradecanol	1664	235.7a	177.8a	528.3a	261.7	45.8a	241.5a	25.1a	27.4a	135.7a	152.2a	***
**Aldehydes and Ketones**												
Hexanal	777	165.6b	0.0b	698.9a	0.0b	132.5b	50.3b	0.0b	0.0b	0.0b	0.0b	***
Furfural	804	2305cd	2766cd	1975cd	1494d	1429d	9163a	6836ab	4501bc	7586a	6702ab	***
Benzaldehyde	925	1302cd	3374a	505de	1907bc	133e	693de	1690bc	1270cd	2330b	1154cd	***
2-Methyltetrahydrothiophen-3-one	952	938.3a	500.3bc	173.1d	540.6bc	0.0d	395.9c	47.2d	89.3d	672.0b	629.3b	***
6-methyl-5-Hepten-2-one	967	261.0de	1280.1ad	1691.5ab	841.5be	47.6e	407.0ce	1356.9ac	2165.6a	16.2e	113.8e	***
Nonanal	1083	248.7ab	238.2ab	0.0b	617.8a	213.3ab	117.2ab	178.3ab	0.0b	87.5ab	31.7ab	*
Benzophenone	1612	101.5ab	17.9b	147.1a	36.4ab	0.0b	44.5ab	21.1b	14.2b	71.5ab	0.0b	**
3,7,11-trimethyl-2,6,10-Dodecatrienal	1730	217.9ab	433.6a	96.1ab	430.8a	0.0b	149.2ab	62.0ab	56.8ab	236.5ab	201.1ab	*
**Terpenoids**												
α-phellandrene	1003	108.5b	112.6b	301.7a	0.0d	0.0d	46.8cd	17.3cd	40.1cd	13.0cd	55.2c	***
o-cymene	1014	122.0ab	101.2ab	158.2a	0.0b	0.0b	31.1ab	14.5ab	19.0ab	0.0b	27.1ab	**
α-terpineol	1171	29.5a	14.1a	65.8a	18.0a	27.6a	42.4a	54.2a	41.2a	31.4a	27.6a	***
β-damascenone	1359	3608.2a	2273.0ab	1579.5ab	1905.1ab	152.3b	1746.3ab	1546.5ab	1350.2ab	346.5b	539.9b	**
β-famesene	1458	913.4ab	2682.7a	367.1b	2770.5a	18.3b	1121.4ab	338.9b	259.2b	992.2ab	824.3ab	***
a-farnesene	1480	211.9b	643.6a	36.9c	515.4ab	0.0c	401.0b	62.5c	30.0c	183.2b	138.1b	***
Nerolidol	1552	4062ac	14,333a	2418bc	11,995ab	15,000c	2297bc	2255bc	1218bc	3525ac	3162bc	**
**Other Compounds**												
1,1-diethoxy-propane	814	0.0c	0.0c	99.9a	0.0c	0.0c	0.0c	0.0c	43.4b	0.0c	0.0c	***
Benzothiazole	1186	266.0a	0.0a	275.7a	324.2a	0.0a	0.0a	0.0a	362.5a	0.0a	0.0a	**

Same letters next to mean values within rows indicate the lack of statistically significant differences at *p* < 0.05, *n* = 3, *ns*—not significant; 0.001= ***; 0.01 = **; 0.05 = *; ^2^ LRI—linear retention index; the amount of compounds was determined semi-quantitatively by measuring the relative peak area of each identified compound, according to the National Institute of Standards and Technology (NIST) database, in relation to that of the internal standard.

## Supplementary Materials

The following are available online at https://www.mdpi.com/2218-273X/10/6/853/s1, Table S1: Details regarding HPLC method for each sugar and glycerol; Table S2: Pearson correlation coefficients between the intensities of the descriptors and the overall note of the brandies. Summary of statistical analyses carried out for results of sensory analysis.
